# Deep Brain Stimulation for Parkinson’s Disease—A Narrative Review

**DOI:** 10.3390/biomedicines13102430

**Published:** 2025-10-05

**Authors:** Rafał Wójcik, Anna Dębska, Karol Zaczkowski, Bartosz Szmyd, Małgorzata Podstawka, Ernest J. Bobeff, Michał Piotrowski, Paweł Ratajczyk, Dariusz J. Jaskólski, Karol Wiśniewski

**Affiliations:** 1 Department of Neurosurgery and Oncology of Central Nervous System, Barlicki University Hospital, Medical University of Lodz, 90-153 Lodz, Poland; anna.debska2@stud.umed.lodz.pl (A.D.); karol.zaczkowski@stud.umed.lodz.pl (K.Z.); bartosz.szmyd@umed.lodz.pl (B.S.); malgorzata.podstawka@stud.umed.lodz.pl (M.P.); ernest.bobeff@umed.lodz.pl (E.J.B.); michal.piotrowski@umed.lodz.pl (M.P.); dariusz.jaskolski@umed.lodz.pl (D.J.J.); karol.wisniewski@umed.lodz.pl (K.W.); 2 Department of Sleep Medicine and Metabolic Disorders, Medical University of Łódz, 92-215 Łódz, Poland; 3 Department of Anesthesiology and Intensive Therapy, Medical University of Lodz, 90-419 Lodz, Poland; pawel.ratajczyk@umed.lodz.pl

**Keywords:** deep brain stimulation, Parkinson’s disease, subthalamic nucleus, dentato-rubro-thalamic tract, tractography

## Abstract

Deep brain stimulation (DBS) is an established neurosurgical treatment for Parkinson’s disease (PD), mainly targeting motor symptoms resistant to pharmacological therapy. This review examines strategies to optimize DBS using advanced anatomical, functional, and imaging approaches. The subthalamic nucleus (STN) remains the principal target for alleviating bradykinesia and rigidity, while recent evidence highlights the dentato-rubro-thalamic tract (DRTt) as an additional promising target, especially for tremor control. Clinical data demonstrate that co-stimulation of both STN and DRTt via electrode electric fields results in superior motor outcomes, including greater reductions in UPDRS-III scores and lower levodopa requirements. The review highlights the use of high-resolution MRI and diffusion tensor imaging tractography in visualizing STN and DRTt with high precision. These methods support accurate targeting and individualized treatment planning. Electric field modelling is discussed as a tool to quantify stimulation overlap with target structures and predict clinical efficacy. Anatomical variability in DRTt positioning relative to the STN is emphasized, supporting the need for patient-specific DBS approaches. Alternative and emerging DBS targets—including the pedunculopontine nucleus, zona incerta, globus pallidus internus, and nucleus basalis of Meynert—are discussed for their potential in treating axial and cognitive symptoms. The review concludes with a forward-looking discussion on network-based DBS paradigms, the integration of adaptive stimulation technologies, and the potential of multimodal imaging and electrophysiological biomarkers to guide therapy. Together, these advances support a paradigm shift from focal to network-based neuromodulation in PD management.

## 1. Introduction

Deep brain stimulation (DBS) is an essential treatment for Parkinson’s disease (PD). DBS modulates abnormal basal ganglia circuitry by disrupting pathological neuronal firing patterns and promoting more physiological network activity. Additional proposed mechanisms include alterations in oscillatory dynamics, induction of synaptic plasticity, and modulation of neurotransmitter release [1,2,3]. In effect DBS reduces motor symptoms, particularly stiffness and bradykinesia, as well as tremor [4]. Additionally, it enables a reduction in oral drug dosage, thereby alleviating drug-related side effects. By restoring more physiological activity in motor circuits, DBS not only alleviates symptoms but also improves patients’ overall quality of life. The mechanism of this treatment involves electrical stimulation of specific areas in the brain, particularly the subthalamic nucleus (STN) [5]. The sustained clinical benefits and relatively low incidence of adverse events associated with DBS underscore its importance as a significant therapeutic modality in the multidisciplinary treatment of PD.

STN serves as the primary target for DBS in PD treatment. While other targets like the internal globus pallidus (GPi) and thalamus are utilized, STN DBS exhibits superior efficacy in alleviating motor symptoms [6,7]. Complications are rare, and hospital stays are usually short, crucial considerations given patients’ advanced age and comorbidities [8]. Qualified neurologists specializing in extrapyramidal diseases oversee patient selection and management pre- and post-procedure. Although DBS is a relatively small-scale intervention, STN targeting requires neurosurgeons to undergo multi-stage training in stereotactic techniques to ensure precision and safety.

Currently, increasing attention is being given to whether stimulation is performed unilaterally or bilaterally. The latest literature review indicates that in PD, unilateral DBS of the STN or GPi improves motor scores by up to ~37%, while bilateral DBS achieves up to ~66% C. Unilateral stimulation yields up to 75% improvement in contralateral symptoms but only up to 28% ipsilaterally, and more than half of patients eventually undergo surgery on the second side due to symptom progression. In essential tremor, unilateral VIM DBS improves hand tremor by up to ~89%, with staged bilateral procedures adding 77–89% contralateral improvement. Axial tremor improves by up to 60% after unilateral VIM DBS, with a further ~60% after bilateral surgery [9]. However, bilateral VIM DBS is more frequently associated with adverse effects such as gait disturbance and dysarthria, whereas STN and GPi stimulation carry comparable risks. Staged procedures are also linked to longer surgical times, higher revision rates, and more complex programming requirements [9].

Stimulating the dentato-rubro-thalamic tract (DRTt) has emerged as a promising strategy for alleviating tremor, particularly in PD patients [10]. This novel approach not only targets Parkinson’s tremor but also shows potential in addressing spontaneous tremor. Recent research has demonstrated notable tremor reduction in patients undergoing stimulation, particularly when the STN was stimulated close to this tract. These findings highlight the growing effectiveness of DBS techniques in managing tremor-related conditions, offering hope for improved therapeutic outcomes in the future.

## 2. Anatomy and Function of Basal Ganglia

### 2.1. Subthalamic Nucleus in PD

**Figure 1:**
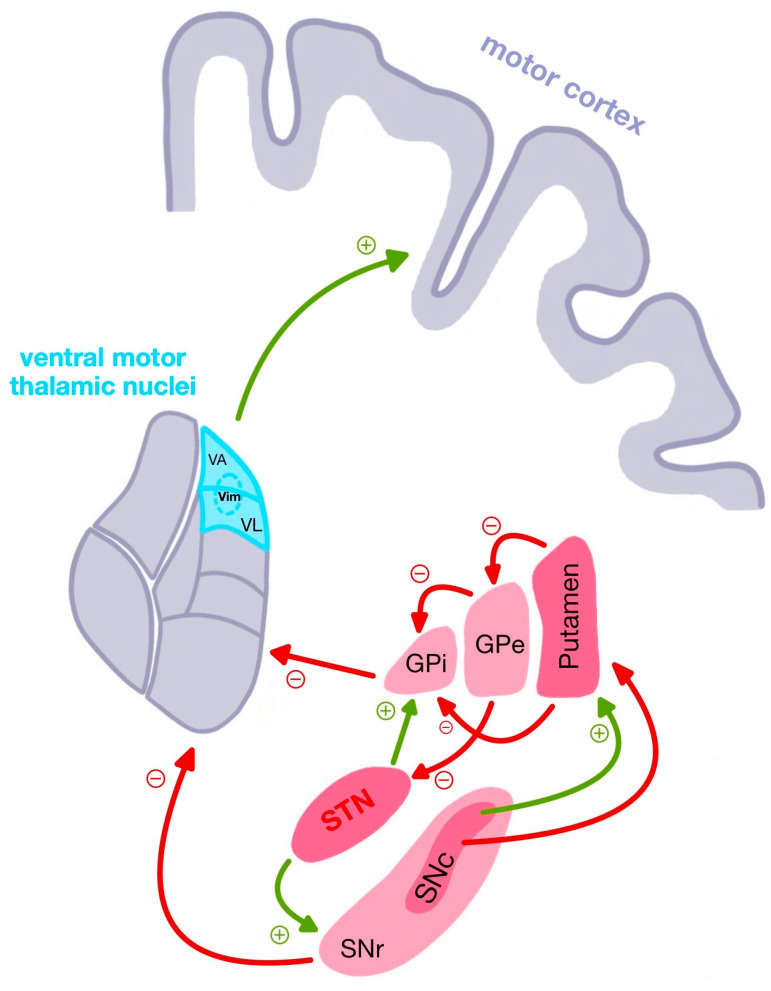
Illustration of the Basal Ganglia Circuitry in Parkinson’s Disease. Legend: GPe—external globus pallidus; GPi—internal globus pallidus; SNc—substantia nigra pars compacta; SNr—substantia nigra pars reticulata; STN—subthalamic nucleus; VA—ventral anterior nucleus; VL—ventral lateral nucleus; VM—ventral medial nucleus.

The STN is a small, glutamatergic structure located within the basal ganglia, interfacing with the external globus pallidus (GPe), GPi, substantia nigra, and thalamus (see Figure 1 and Table 1). Under physiological conditions, the STN plays a critical role in the indirect pathway of basal ganglia motor control, exerting excitatory influence on the GPi and substantia nigra pars reticulata (SNr), which then inhibit thalamocortical motor drive. In PD, dopaminergic neuronal loss in the substantia nigra pars compacta (SNc) leads to overactivation of the STN, resulting in increased inhibitory output from GPi/SNr to the motor cortex and suppression of voluntary movements [11,12].

The pathological overactivity of the STN contributes not only to bradykinesia and rigidity but may also exacerbate abnormal firing patterns and synchronization within basal ganglia-thalamocortical circuits. This has formed the basis for the therapeutic application of DBS targeting the STN (STN-DBS), which modulates aberrant STN activity, leading to significant improvement in motor symptoms [11,13]. However, variability in STN-DBS outcomes remains, with neuroimaging evidence suggesting that patients with greater preoperative brain atrophy, reduced SNc volume, and enlarged ventricles have poorer motor improvement. These structural changes, especially in combination with lower executive function scores, point to the influence of subclinical dementia on DBS efficacy [11].

### 2.2. Dentato-Rubro-Thalamic Tract and Its Role in Motor Modulation

DRTt is a cerebellar efferent pathway that begins in the dentate nucleus of the cerebellum, ascends via the superior cerebellar peduncle, a part of them decussates at the midbrain, and both with non-decussated projects to the ventral lateral thalamus via pathways passing anteriorly to the red nucleus (RN). It comprises both decussating and non-decussating fibers, which terminate in different thalamic nuclei [10,14].

Functionally, the DRTt is central to the cerebello-thalamo-cortical loop, a system known to coordinate fine motor control, tremor suppression, and movement precision. While the basal ganglia have long been emphasized in PD pathogenesis, there is growing recognition of the cerebellum’s role, particularly in resting tremor, which is often resistant to standard basal ganglia-based therapies [15,16].

### 2.3. Simultaneous STN and DRTt Stimulation in PD

Recent evidence supports the clinical relevance of the DRTt in PD treatment. In a study by Wiśniewski et al. (2024) [10], simultaneous stimulation of the STN and DRTt, as determined by the overlap of the electrode’s electric field (EF) with both structures, resulted in significantly better motor outcomes than stimulation limited to the STN. Patients with EF overlapping both targets had more than 50% improvement in UPDRS-III scores in most cases. The distance between the DRTt and the EF emerged as the most robust predictor of clinical efficacy (AUC > 0.9), suggesting a synergistic effect when both the STN and DRTt are modulated.

Anatomical studies using white matter dissection (Klingler technique) confirmed substantial interindividual variability in the STN–DRTt relationship. These findings emphasize the importance of personalized DBS targeting, especially in patients with predominant tremor symptoms, where extending the stimulation field to the DRTt may yield additional benefit [10].

### 2.4. Implications for DBS Strategy and Future Directions

Together, the STN and DRTt represent complementary targets in the management of PD motor symptoms. While the STN remains the primary focus for addressing bradykinesia and rigidity, the DRTt offers a promising avenue for enhancing tremor control, particularly when conventional targeting is suboptimal. Incorporating preoperative volumetric MRI and diffusion tensor imaging tractography into DBS planning may improve patient selection and electrode placement by accounting for both neurodegenerative burden and tract proximity.

Moreover, identifying biomarkers such as atrophy ratio, SNc and thalamic volumes, and cognitive scores (e.g., FAB) may help anticipate long-term outcomes and tailor stimulation strategies to individual neuroanatomy and symptom profiles [11,17].

### 2.5. Alternative and Adjunct DBS Targets: PPN, Zona Incerta, and Globus Pallidus Internus

While the STN and DRTt remain central to motor symptom modulation in PD, other deep brain structures have been identified as valuable targets, particularly for addressing symptoms that are resistant to STN-DBS. One such structure is the pedunculopontine nucleus (PPN) in the caudal mesencephalic tegmentum near the superior cerebellar peduncle. The PPN is part of the mesencephalic locomotor region (MLR) and has extensive connections with the basal ganglia, thalamus, cerebellum, and spinal cord. It is critically involved in gait regulation, postural control, and arousal mechanisms. As such, low-frequency PPN stimulation has been proposed to alleviate axial symptoms such as freezing of gait (FOG), falls, and postural instability, which are often refractory to STN or GPi DBS. Preliminary studies have shown improvements in gait initiation and reduced fall frequency with unilateral or bilateral PPN-DBS [18,19,20]. However, clinical results have been inconsistent, likely due to anatomical variability, technical challenges in targeting, and lack of standardized stimulation parameters [21].

The zona incerta (ZI), a poorly defined region situated dorsomedially to the STN and ventral to the thalamus, is another promising DBS target, particularly in the treatment of tremor-dominant PD and essential tremor. The caudal ZI (cZI) is considered part of the subthalamic area rich in inhibitory (GABAergic) neurons and adjacent to the DRTt, making it an effective site for tremor control. Clinical studies have demonstrated that stimulation of the ZI, especially the cZI, can result in greater tremor suppression compared to stimulation of the ventral intermediate nucleus (Vim) of the thalamus or the STN, while also producing fewer stimulation-induced side effects [22,23,24]. The anatomical proximity of the ZI to multiple sensorimotor pathways suggests a multifaceted mechanism of action, potentially involving cerebellothalamic, vestibular, and basal ganglia circuits [25].

The GPi has long been established as a primary DBS target, especially in dystonia and Parkinsonian motor complications. The GPi is a major output nucleus of the basal ganglia, exerting inhibitory control over the thalamus. In PD, the overactivity of the GPi contributes to motor inhibition and dysregulation of thalamocortical drive. DBS of the GPi is particularly effective in reducing levodopa-induced dyskinesias (LID), offering sustained relief while minimizing psychiatric and cognitive adverse effects more common with STN stimulation [13,26]. Additionally, GPi-DBS tends to allow continued dopaminergic therapy, offering better motor stability in some patients compared to STN-DBS, which typically leads to more significant reductions in levodopa equivalents. A randomized controlled trial by Odekerken et al. (2013) [26] found that while both GPi and STN stimulation significantly improved motor function, GPi-DBS was associated with fewer mood and cognitive side effects, making it a preferred option in older patients or those with preexisting cognitive deficits.

These alternative DBS targets—PPN, ZI, and GPi—highlight the evolving understanding of PD as a disorder that affects distributed motor and non-motor networks beyond the classic nigrostriatal pathway. Their selective application, guided by patient-specific symptoms, cognitive status, and imaging biomarkers, may enhance clinical outcomes and support the development of tailored, network-based stimulation strategies in PD.

### 2.6. Ventral Intermediate Nucleus Stimulation in PD

Vim of the thalamus has historically been the primary target for DBS in patients with tremor-dominant PD. Anatomically, the Vim receives major afferents from the dentate nucleus of the cerebellum via the DRTt and projects to the primary motor cortex, thereby playing a central role in the generation and propagation of tremor-related activity. Vim-DBS was first reported as an effective method for tremor suppression by Benabid et al. [27] in the early 1990s, laying the foundation for modern DBS therapy [27]. In multiple studies, Vim stimulation has demonstrated robust efficacy in reducing resting, postural, and intention tremor in PD, particularly in patients for whom tremor is the predominant and most disabling motor symptom [28,29].

Unlike STN or GPi stimulation, however, Vim-DBS does not significantly improve bradykinesia, rigidity, or axial symptoms, and has minimal influence on levodopa-induced dyskinesias. Therefore, it is not recommended as a first-line target in patients with complex or generalized motor fluctuations. Additionally, Vim-DBS does not permit a substantial reduction in dopaminergic medications, a key benefit of STN-DBS [5]. Long-term follow-up has shown sustained tremor control in many cases. Still, habituation or effect waning can occur over time, likely due to disease progression or tolerance to chronic stimulation [30,31]. Adverse effects are generally mild and stimulation-related, including dysarthria, paresthesias, or limb ataxia, particularly if current spreads to adjacent sensory thalamic nuclei.

Nevertheless, Vim-DBS remains an important therapeutic option in older patients, individuals with significant cognitive impairment, or those contraindicated for STN or GPi DBS, as it carries a lower risk of cognitive and behavioral complications. With advances in tractography-based targeting, efforts are underway to better localize the Vim and its connections with the DRTt to optimize outcomes and minimize side effects [15]. Overall, while no longer the dominant DBS target in PD, Vim stimulation retains an important niche in the personalized treatment of tremor-predominant phenotypes.

### 2.7. Post-Subthalamic Area in PD

The post-subthalamic area (PSA), encompassing the cZI, prelemniscal radiation (Raprl), and neighboring fiber-rich regions, is increasingly recognized as a highly effective DBS target for managing tremor-predominant PD, as well as essential tremor (ET) and other movement disorders. Situated posterior and inferior to the STN the PSA offers direct access to cerebellothalamic projections, particularly the DRTt—a pathway critically involved in tremor genesis and control [32]. Unlike STN or Vim stimulation, PSA-DBS has demonstrated superior tremor suppression across rest, postural, and kinetic tremors, often at lower amplitudes and with fewer side effects (dysarthria, ataxia, paresthesias) due to its greater distance from sensory relay nuclei [22,24,33].

Anatomically, the PSA is uniquely positioned as a convergence zone for motor-related white matter tracts, including the DRTt, pallidothalamic fibers, and mesencephalic locomotor connections. This strategic location makes it a valuable target for modulating both basal ganglia and cerebellar circuits involved in motor control [34]. Studies utilizing directional leads and diffusion tractography have refined PSA targeting, demonstrating that the most effective contact sites are located near the posterior STN–ZI interface or within the prelemniscal radiation, where stimulation fields intersect with the DRTt [24,35]. Functionally, PSA-DBS can offer tremor control even in patients unresponsive to STN or Vim-DBS, suggesting an independent mechanism of action through cerebellothalamic modulation [34].

While STN-DBS remains superior for managing bradykinesia and rigidity, PSA stimulation is particularly advantageous in patients with severe or medication-resistant tremor, including cases with minimal levodopa responsiveness. Its benefits have also extended to multi-target approaches, where PSA contacts are combined with STN stimulation to address complex symptomatology [36,37]. However, challenges remain, including variability in PSA anatomy, lack of consensus on optimal coordinates, and difficulty distinguishing the cZI from surrounding structures on standard MRI. Future directions include tractography-guided implantation, connectomic mapping, and integration into adaptive DBS systems to exploit the dynamic properties of the PSA [38,39].

### 2.8. Nucleus Basalis of Meynert Stimulation in PD Dementia

PD dementia (PDD) and mild cognitive impairment (MCI) are common non-motor complications of PD, affecting up to 80% of patients over the disease course. These cognitive deficits are often characterized by impairments in attention, executive function, memory, and visuospatial processing, and are closely linked to cholinergic degeneration within the nucleus basalis of Meynert (NBM). The NBM, located in the basal forebrain, is the principal source of cholinergic projections to the neocortex, and its progressive degeneration is a hallmark of both PDD and Alzheimer’s disease. Given the limited efficacy of pharmacological cholinesterase inhibitors, DBS of the NBM has emerged as a novel experimental strategy to restore cholinergic tone and ameliorate cognitive symptoms.

Initial case studies and small trials have explored low-frequency NBM-DBS in PD patients with MCI or early-stage dementia, showing modest improvements in attention, arousal, and executive processing, without significant adverse effects [40,41]. The stimulation is typically bilateral and targets the Ch4 cell group, which sends widespread cortical projections. In a double-blind study by Gratwicke et al. [40], NBM-DBS in patients with PDD resulted in variable cognitive responses, suggesting that patient selection and precise targeting may be critical determinants of efficacy. Moreover, imaging and electrophysiological studies have shown that NBM-DBS can enhance cortical EEG desynchronization and increase cholinergic network activity, potentially supporting cognitive function [42].

Despite these early findings, NBM stimulation remains investigational, and its clinical benefit in PD requires further validation through larger, controlled trials. The heterogeneity of cognitive impairment in PD and the anatomical variability of the NBM pose additional challenges to consistent outcomes. Nevertheless, NBM-DBS represents a promising neuromodulatory approach for addressing non-motor symptoms in PD, particularly in the context of a multi-target DBS strategy aimed at both motor and cognitive domains.

### 2.9. Cuneiform Nucleus in PD

The cuneiform nucleus (CnF), located dorsolaterally to the PPN within MLR, has recently gained attention as a promising DBS target for PD, particularly for patients suffering from FOG and other axial motor symptoms refractory to conventional STN or GPi stimulation. While the PPN has been the historical focus for DBS targeting within the MLR, inconsistent outcomes have prompted a shift toward the CnF due to its more consistent anatomical definition and predominantly glutamatergic output, which provides strong excitatory input to the medullary reticulospinal circuits responsible for locomotor drive [43,44,45]. Preclinical studies using optogenetics and electrical stimulation in 6-OHDA lesioned rodents have demonstrated that activating the CnF can significantly improve locomotor initiation, step length, and gait coordination, even in dopamine-depleted conditions that model PD [46]. Compared to the PPN, the CnF has shown greater reliability in eliciting locomotor activity in decerebrate animal models, suggesting a more robust and direct influence on central pattern generators [44,47]. In humans, intraoperative electrophysiological recordings and pilot stimulation studies suggest that targeting the CnF may alleviate gait freezing and improve motor stability, particularly in patients with severe axial symptoms unresponsive to STN-DBS [48,49]. Additionally, imaging and neuropathological studies show early degeneration of CnF neurons in PD, linking its dysfunction to the emergence of non-dopaminergic gait disturbances [50]. Connectomic analyses further reveal that the CnF is part of a broader brainstem–cortical locomotor network, and effective CnF-DBS may modulate this circuitry to restore gait control [36]. Despite its promise, the CnF remains understudied in clinical DBS research, and larger, controlled trials are needed to validate its efficacy, define optimal stimulation parameters, and assess long-term outcomes. Nevertheless, its unique anatomical and functional characteristics suggest that CnF-DBS could become a critical component of network-based, multi-target neuromodulation strategies for advanced PD [51].

### 2.10. Centromedian–Parafascicular Complex in PD

The centromedian–parafascicular (CM-Pf) complex of the thalamus, part of the intralaminar nuclei, plays a key modulatory role in motor, cognitive, and attentional processing and has been increasingly studied as a potential target in DBS for PD. These nuclei form dense reciprocal projections with the basal ganglia, especially the striatum, and contribute to the thalamostriatal pathway, which is crucial for action selection, motor planning, and behavioral flexibility [52,53]. In PD, degeneration of CM-Pf neurons has been consistently documented, correlating with non-motor symptoms such as apathy, attention deficits, and executive dysfunction, as well as with aspects of motor freezing and response inhibition [54,55]. Functional studies have shown that the CM-Pf is activated during tasks requiring salience detection, error monitoring, and responses to novelty—functions often impaired in PD [56]. These functions suggest a potential benefit of modulating CM-Pf activity through DBS to improve cognitive and behavioral symptoms, in addition to motor outcomes.

Though traditionally not targeted in routine PD DBS, the CM-Pf has shown promise in case series and pilot studies, especially in patients with axial symptoms, tremor refractory to STN or Vim stimulation, or neuropsychiatric features. For example, CM-Pf stimulation has been reported to modulate cortical–striatal rhythms, reduce levodopa-induced dyskinesias, and even improve tremor resistant to levodopa or conventional DBS targets [57,58,59]. Furthermore, because CM-Pf is anatomically and functionally integrated into both motor and limbic circuits, stimulation may offer dual benefit for motor and neuropsychiatric symptoms [60]. Recent connectomic and tractography studies suggest that CM-Pf-DBS may modulate distributed basal ganglia-thalamocortical loops, aligning with the network-based paradigm of neuromodulation in PD [61]. While more research is needed, particularly randomized controlled trials and long-term follow-up studies, CM-Pf-DBS represents a promising adjunctive or alternative strategy in the personalized treatment of complex Parkinsonian phenotypes.

### 2.11. Hypothalamic Stimulation for Autonomic Dysfunction in PD

The hypothalamus, particularly its lateral and posterior nuclei, has been proposed as a rare and experimental target for DBS in PD patients suffering from severe autonomic dysfunctions. Autonomic symptoms—including orthostatic hypotension, thermoregulatory abnormalities, urinary incontinence, and gastrointestinal dysmotility—are common in advanced PD and often respond poorly to pharmacological therapies. The posterior hypothalamus (PH) plays a central role in cardiovascular and thermoregulatory regulation via descending projections to the brainstem and spinal cord, while the lateral hypothalamic area (LHA) is involved in feeding behavior, arousal, and energy balance [62,63]. Historical reports on hypothalamic stimulation have primarily focused on cluster headache and intractable hypertension, but incidental autonomic effects observed in these contexts have prompted interest in its potential utility for PD-related dysautonomia [64].

Preclinical studies have demonstrated that electrical stimulation of the PH or LHA in animal models can modulate sympathetic tone, blood pressure, and heart rate variability [65]. In PD, a few small case series and investigational trials have reported modest improvements in orthostatic hypotension and bladder control following hypothalamic DBS, but the data remain sparse and uncontrolled [66]. Moreover, the proximity to critical nuclei involved in arousal and respiratory function poses substantial surgical and ethical challenges, limiting its broader application. As such, hypothalamic DBS remains a largely investigational approach, with its use confined to highly selected cases or within clinical trials aimed at exploring autonomic symptom relief in PD. Future research using advanced imaging, autonomic biomarkers, and closed-loop modulation may help refine its application and clarify patient selection criteria.

### 2.12. Medial Forebrain Bundle in PD

The medial forebrain bundle (MFB) is a major dopaminergic and limbic pathway that connects the ventral tegmental area (VTA) with the nucleus accumbens, hypothalamus, and prefrontal cortex, playing a central role in reward processing, motivation, mood regulation, and drive-related behavior. In PD, degeneration of the dopaminergic system affects not only the motor circuits of the nigrostriatal pathway but also the mesolimbic and mesocortical pathways involving the MFB, which contributes to non-motor symptoms such as apathy, depression, and anhedonia [67,68]. DBS targeting the MFB has been investigated primarily in patients with treatment-resistant depression, where stimulation has shown rapid and robust mood improvements [69]. Given the overlap between motivational deficits in depression and Parkinson-related apathy or mood disorders, MFB-DBS has emerged as a potential investigational approach for addressing severe non-motor symptoms in PD, particularly when traditional pharmacological strategies fail.

Although clinical experience with MFB-DBS in PD is very limited, early case reports and pilot studies suggest that stimulation of the superolateral branch of the MFB may improve mood, drive, and emotional engagement in PD patients with refractory apathy or depression [70,71]. Functional imaging studies indicate that MFB stimulation modulates a broad network involving the ventral striatum, orbitofrontal cortex, and anterior cingulate cortex, supporting its relevance to affective processing. However, challenges remain regarding targeting precision, side-effect management, and the long-term effects of chronic stimulation. As such, MFB-DBS in PD remains highly experimental, with ongoing studies required to determine its safety, efficacy, and appropriate patient selection criteria. Nonetheless, it represents a promising direction in the treatment of disabling non-motor features that are often under-addressed in standard DBS paradigms focused solely on motor improvement.

## 3. Visualization Methods for STN and DRTt

### 3.1. Visualization Methods for the Subthalamic Nucleus and Dentato-Rubro-Thalamic Tract (DRTt)

**Figure 2:**
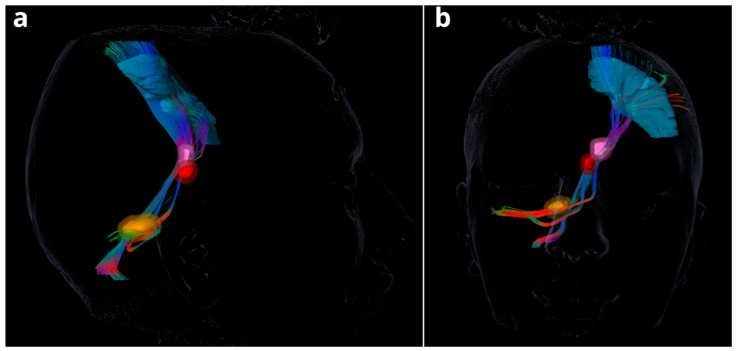
MRI Tractography of the Dentato-Rubro-Thalamic Tract (DRTt): (**a**) lateral view; (**b**) anterior–posterior view. DRTt starts in the dentate nucleus then courses through the superior cerebellar peduncle, and projects to the contralateral red nucleus and ventrolateral thalamic nuclei. Additionally, from the thalamus, fibers connect further to the primary motor cortex and premotor areas. Legend: Red—fibers running along the left–right (x) axis; Green—fibers running along the anterior–posterior (y) axis; Blue—fibers running along the superior–inferior (z) axis; while mixed colors indicate fibers with components in more than one direction, reflecting oblique trajectories.

**Figure 3:**
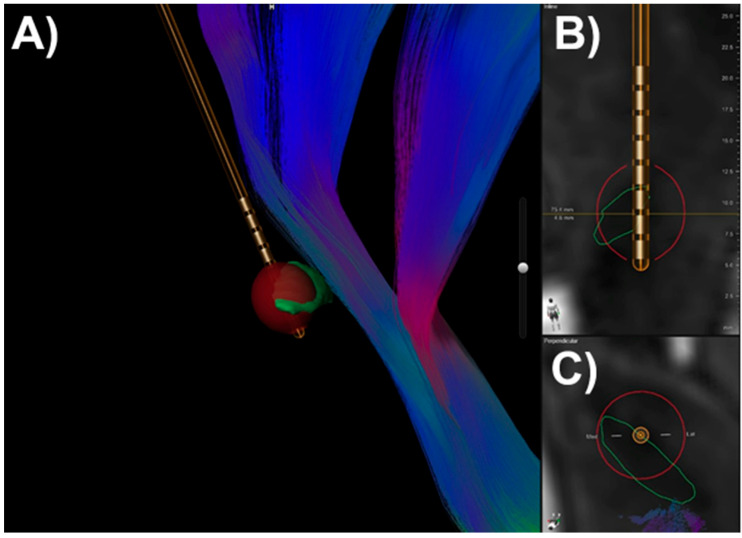
The illustration of the dentato-rubro-thalamic tract, as well as the position of the electrode within the STN, along with the electric field: (**A**) 3D reconstruction showing the electrode (gold) in relation to the DRTt (multicolored fibers) and the subthalamic nucleus (STN, red), with the modeled electric field (green); (**B**) coronal view illustrating the electrode trajectory and electric field overlay within the STN; (**C**) axial view demonstrating the spatial relationship between the electrode, the electric field, and the DRTt fibers.

The success of DBS in PD relies heavily on accurate visualization and targeting of key anatomical structures such as the STN and DRTt (see Figure 2). The STN is a small, lens-shaped structure situated ventral to the thalamus and dorsal to the substantia nigra, and it plays a central role in basal ganglia circuitry (see Figure 3). Its visualization is typically achieved using high-resolution structural MRI, with T2-weighted, susceptibility-weighted imaging (SWI), or FGATIR (Fast Gray Matter Acquisition T1 Inversion Recovery) sequences providing the best contrast to delineate the STN from adjacent nuclei [72,73]. For targeting in clinical practice, the use of 3T MRI is common, and co-registration with postoperative CT allows accurate mapping of electrode location in relation to the nucleus.

In contrast, the DRTt—an essential component of the cerebello-thalamo-cortical motor pathway—is a white matter fiber tract that cannot be visualized directly on conventional structural MRI. Instead, its visualization relies on diffusion-weighted imaging (DWI) and tractography, which reconstructs fiber pathways based on the anisotropic diffusion of water molecules along axonal tracts. Deterministic or probabilistic fiber tracking algorithms are applied to preoperative DWI data to reconstruct the DRTt trajectory from the dentate nucleus of the cerebellum, through the superior cerebellar peduncle, passing anterior to the RN, and terminating in the ventrolateral thalamic nuclei [15,38]. To ensure anatomical validity, regions of interest (ROIs) are typically placed on known landmarks such as the dentate nucleus, RN, and thalamus, guided by anatomical atlases.

Software platforms like Brainlab, Lead-DBS, and Guide™ XT have become standard tools in clinical research and surgical planning. These platforms allow co-registration of multimodal imaging data and estimation of the volume of tissue activated (VTA), which can be overlapped with reconstructed tracts to assess whether stimulation fields intersect the DRTt and/or STN [10]. Importantly, recent studies have demonstrated that patients whose electrode stimulation fields overlap both the STN and the DRTt experience superior motor outcomes, suggesting that individualized tractography-based targeting may enhance DBS efficacy [10,74].

To validate tractography findings, postmortem white matter dissection using the Klingler technique remains the gold standard. This method involves formalin fixation, deep freezing, and layer-by-layer dissection of brain tissue to expose white matter tracts. Studies using this technique have confirmed the anatomical variability of the DRTt, including non-decussating fibers, and their proximity to the STN—findings which corroborate in vivo imaging data and highlight the need for patient-specific surgical planning [14,75].

In sum, the integration of advanced MRI, tractography, and image-guided planning tools, alongside anatomical validation, enables precise, patient-specific DBS targeting of both gray and white matter structures. This multimodal approach is particularly valuable in complex cases of PD, where maximizing therapeutic benefit while minimizing side effects hinges on individualized targeting of both the STN and DRTt.

### 3.2. Calculation of the Electric Field in DBS and Its Anatomical Correlation with STN and DRTt

In the field of functional neurosurgery, accurate modeling of the EF generated by DBS electrodes is essential for understanding and optimizing stimulation outcomes. The EF defines the VTA—the spatial region within which axons and neuronal soma are modulated by the delivered electrical impulses. The EF is not a fixed anatomical entity, but a dynamic and patient-specific volume influenced by both electrode placement and stimulation parameters. In PD, the proximity of the EF to key motor pathways—especially the STN and the DRTt—has been shown to significantly impact clinical efficacy, particularly in the control of tremor, rigidity and bradykinesia.

The procedure for calculating the EF begins with co-registration of preoperative 3T MRI (including structural and diffusion-weighted sequences) and postoperative computed tomography to localize DBS leads in the patient’s native space. Dedicated software platforms (e.g., Brainlab Elements, Guide™ XT, Lead-DBS) reconstruct the electrode trajectory in 3D and identify the active contacts. Parameters required for EF modeling include pulse width (µs), amplitude (V or mA), frequency (Hz), and the specific electrode model and configuration. These inputs are then processed using finite element modeling to simulate the spread of the electrical field through the brain tissue, which is modeled with inhomogeneous conductivity values (typically between 0.1 and 0.3 S/m depending on the tissue type) [76].

The resulting EF is visualized as a volumetric shell or gradient map surrounding the electrode contact, often thresholded at activation levels between 0.2 and 0.4 V/mm, which correspond to the empirically derived threshold for axonal depolarization. Once generated, the EF is overlaid on individual neuroanatomical reconstructions: the STN segmented from high-resolution T2 or FGATIR imaging, and the DRTt reconstructed using diffusion tractography with 64-direction DWI and deterministic or probabilistic tracking algorithms [15,38].

To assess the influence of EF on these structures, researchers calculate distance metrics such as:

- The shortest distance between the EF boundary and the centerline or edge of the DRTt
- The degree of volumetric overlap between the EF and the anatomical extent of the STN or DRTt
- The percentage of DRTt fibers within the EF

These metrics provide a quantifiable framework to predict clinical response.

Moreover, the integration of EF modeling into DBS workflows supports the development of patient-specific stimulation strategies, allowing clinicians to adjust contact selection and parameters to maximize benefit while minimizing side effects such as dysarthria or paresthesias. As computational tools evolve, real-time EF simulation may further refine adaptive DBS systems, where stimulation dynamically responds to physiological feedback.

## 4. Clinical and Anatomical Results

### 4.1. Clinical and Anatomical Results of STN and DRTt Stimulation in PD

DBS of the STN is an established treatment for motor fluctuations in advanced PD, providing significant improvements in bradykinesia, rigidity, and, to a variable extent, tremor [77,78]. However, variability in clinical outcomes has led researchers to explore the contribution of adjacent fiber tracts, such as the DRTt, particularly in tremor-dominant or medication-refractory patients.

In the study by Wiśniewski et al. (2024) [10], patients whose EFs overlapped both the STN and DRTt showed greater improvements in UPDRS-III motor scores, and the distance between EF and DRTt emerged as the most powerful predictor of motor benefit, with receiver operating characteristic (ROC) curve AUC values > 0.9. These findings suggest that the functional connectivity and anatomical proximity of the DRTt to the STN can be harnessed through careful surgical targeting and postoperative programming. These findings are consistent with prior reports by Coenen et al. (2011) [15], who demonstrated that intentional targeting of the DRTt can significantly reduce tremor severity, even in patients unresponsive to standard thalamic or STN targets. Moreover, the group with STN–DRTt EF overlap also required lower stimulation amplitudes and exhibited greater reductions in levodopa equivalent daily dose (LEDD), indicating a more efficient and durable therapeutic effect. These findings support the concept that co-stimulation of cerebellothalamic fibers may synergize with basal ganglia modulation to achieve more robust motor benefits [79,80].

### 4.2. Anatomical Correlation

The DRTt, a major cerebellothalamic output pathway, originates in the dentate nucleus, ascends through the superior cerebellar peduncle, and travels anterior to RN to reach the ventrolateral thalamus [14,38]. Its proximity to the dorsolateral STN explains why traditional STN-DBS may inadvertently stimulate cerebellar fibers, particularly when high stimulation amplitudes or directional leads are used [81].

Diffusion-weighted imaging (DWI) with deterministic or probabilistic tractography has enabled preoperative visualization of the DRTt. In the Wiśniewski study [6], the DRTt was successfully reconstructed in all patients and showed individual variability in trajectory, emphasizing the importance of patient-specific tractography. Importantly, in cadaveric dissections using the Klingler technique, DRTt fibers were identified within 2.4 mm vertically and ~4.3 mm obliquely from the STN, confirming the anatomical plausibility of functional co-stimulation [75].

These findings are supported by functional imaging studies showing that tremor control correlates with connectivity between DBS contacts and motor cerebellar networks, particularly via the DRTt [82]. Additionally, Fenoy and Schiess demonstrated that direct targeting of the DRTt using tractography-guided DBS led to sustained tremor control in PD patients, especially when traditional STN or thalamic stimulation had failed [74].

## 5. Clinical Implications and Future Directions

### 5.1. Implications for Optimizing DBS in PD

Emerging evidence suggests that the simultaneous stimulation of the STN and the DRTt may represent a promising direction in the neuromodulation paradigm for PD. While traditional approaches to DBS have focused on isolated targets such as the STN or the GPi, recent observations indicate that tremor relief can be enhanced when stimulation also modulates key white matter tracts like the DRTt. However, current findings remain preliminary and further studies are needed to clarify the role of this pathway [10,15,74].

The integration of advanced neuroimaging techniques, including tractography-based targeting and EF modeling, has enabled a more nuanced understanding of the spatial relationships between stimulation fields and symptom-relevant structures [76,81]. In patients where the EF overlapped both STN and DRTt, greater improvements in UPDRS-III motor scores, lower stimulation thresholds, and larger reductions in levodopa equivalent daily dose (LEDD) were consistently observed [10]. These findings underscore the importance of shifting from static, coordinate-based targeting to dynamic, patient-specific targeting dynamic strategies guided by anatomical variability and functional connectivity.

The application of directional leads allows clinicians to sculpt current fields to preferentially stimulate the DRTt while avoiding side effects linked to adjacent sensory or associative regions [82]. In tandem, adaptive DBS systems, which respond to real-time neurophysiological feedback, may be tailored to activate the STN–DRTt interface during periods of symptom exacerbation—further refining network-based neuromodulation [83].

### 5.2. Future Research Directions

To validate and expand upon these findings, multiple avenues of future research are warranted:

1. Large-scale randomized controlled trials (RCTs) are necessary to compare traditional STN-DBS against strategies that intentionally co-stimulate the DRTt. Stratification based on motor phenotype (e.g., tremor-dominant vs. akinetic-rigid) and cognitive baseline would clarify which subgroups derive the most benefit [77,78].
2. Standardization of tractography protocols remains a priority. While many studies successfully reconstruct the DRTt using diffusion-weighted imaging (DWI), variability in region-of-interest (ROI) placement and algorithm selection limits reproducibility. Collaborative efforts to define validated tractography atlases for DRTt and STN would enhance targeting accuracy [38,81].
3. Quantitative modeling of EF–DRTt interaction is needed. Future studies should define the minimum EF overlap or distance thresholds predictive of motor benefit, integrating computational models with clinical outcomes [10,76].
4. Functional imaging and connectomic profiling may identify individual biomarkers predictive of DBS success. For example, resting-state fMRI or structural connectomics could guide electrode placement based on symptom-specific connectivity maps [72,82].
5. Electrophysiological studies could identify DRTt-specific oscillatory signatures or biomarkers of effective stimulation, potentially useful for real-time control in adaptive DBS systems [83].
6. Exploring the integration of DRTt stimulation with non-motor DBS targets, such as the NBM, may benefit patients with cognitive decline or PD dementia [40,41].
7. Finally, continued use of white matter dissection techniques, such as the Klingler method, remains essential to validate imaging-derived models of the DRTt and to understand inter-individual anatomical variability [14,75].

Together, these findings support a move toward network-based DBS strategies in PD, recognizing that effective motor symptom control often involves coordinated modulation of both nuclei and fiber tracts. By integrating tractography, EF modeling, and connectomic insights into both surgical planning and postoperative programming, clinicians can personalize therapy to optimize outcomes. Continued research will be essential to refine these approaches, develop predictive biomarkers, and expand the clinical utility of STN–DRTt dual-target stimulation.

## 6. Critical Considerations

Although STN-DBS remains the gold standard for advanced PD, the evidence base has important limitations. Many clinical trials demonstrating its efficacy are restricted by small sample sizes, heterogeneous inclusion criteria, and limited follow-up, which complicate direct comparisons across studies. Moreover, variability in outcomes—especially in patients with cognitive impairment or brain atrophy—suggests that comorbid neurodegenerative processes can attenuate treatment benefit, a factor often underreported in early trials. Similarly, while the DRTt has emerged as a promising adjunct target for tremor, most supporting studies rely on tractography reconstructions and non-randomized cohorts. Diffusion tractography itself is prone to methodological variability, raising concerns about reproducibility and the validity of fiber localization across centers. The apparent synergistic effect of simultaneous STN and DRTt stimulation is encouraging, but it remains based on small observational series; whether this translates into long-term superiority over conventional STN-DBS has yet to be established. Finally, controversies persist regarding whether tremor suppression in STN-DBS results from direct modulation of the STN or inadvertent co-stimulation of adjacent cerebellothalamic fibers, underscoring the need for mechanistic studies that integrate imaging, physiology, and clinical outcomes.

## 7. Conclusions

### 7.1. Summary and Clinical Significance of STN and DRTt Stimulation in PD

Recent evidence demonstrates that optimized DBS for PD involves not only the modulation of the STN—a long-established target—but also the DRTt, a cerebellothalamic fiber pathway critical for tremor control. Clinical outcome analyses indicate that patients whose EF overlaps both the STN and DRTt experience superior motor improvements, including greater reductions in UPDRS-III scores and levodopa equivalent daily doses, compared to those with STN-only stimulation [6,34]. Furthermore, distance between the EF and DRTt emerged as a strong predictor of therapeutic response, with ROC AUC values exceeding 0.9, highlighting the importance of spatial proximity in effective DBS outcomes.

Anatomically, the DRTt lies within a few millimeters of the dorsolateral STN, making possible for conventional STN-targeted leads to incidentally or intentionally engage both structures, especially with high-resolution targeting and directional leads. Validation through diffusion tractography, finite element modeling, and cadaveric dissection confirms this proximity and supports the potential for network-based modulation [14,15,75]. These findings reinforce a more integrative approach to DBS, acknowledging the interplay between basal ganglia and cerebellothalamic circuits.

### 7.2. Clinical and Research Implications

For clinical practice, these insights advocate for the adoption of patient-specific DBS planning that includes: (1) Preoperative tractography to map individual DRTt trajectories; (2) EF modeling to optimize stimulation overlap with symptom-relevant targets; and (3) Directional leads and adaptive DBS systems to fine-tune current spread for maximal efficacy.

In research, future directions should include prospective randomized trials, standardized imaging protocols, and the exploration of functional biomarkers that can guide both targeting and programming. These findings lay the groundwork for a paradigm shift in DBS therapy—moving from focal nucleus-based stimulation to personalized, network-centered neuromodulation, potentially improving both motor and non-motor outcomes in PD.

This indicates the potential significance of these findings for clinical practice and further research on DBS therapy.

## Figures and Tables

**Table 1:** Comparative summary of deep brain stimulation targets and symptom control in Parkinson’s disease.

Target	Circuit/Role	Main Symptoms Improved	Clinical Advantages	Limitations/Considerations
**Subthalamic Nucleus**	Basal ganglia indirect pathway; excitatory drive to GPi/SNr	Bradykinesia, rigidity, tremor (variable)	Allows LEDD reduction, broad motor benefit	Cognitive/mood side effects, variable tremor control
**Globus Pallidus Internus**	Major output nucleus of basal ganglia; inhibitory control of thalamus	Dyskinesias, motor fluctuations	Fewer cognitive/psychiatric side effects; good for older patients	Less LEDD reduction, less effective for tremor
**Dentato-Rubro-Thalamic Tract**	Cerebello-thalamo-cortical loop	Tremor, fine motor control	Tremor suppression, synergistic with STN	Visualization requires tractography; individual variability
**Ventral Intermediate Nucleus**	Thalamic relay of DRTt fibers to cortex	Tremor (resting, postural, intention)	Robust tremor suppression, low cognitive risk	No effect on rigidity/bradykinesia; habituation over time
**Post-Subthalamic Area/Caudal Zona Incerta**	White matter convergence zone incl. DRTt	Tremor (all types)	Superior tremor control, lower side effects vs. Vim	Anatomical variability, targeting challenges
**Pedunculopontine Nucleus**	Mesencephalic locomotor region; gait/posture control	Freezing of gait, falls, postural instability	May improve axial symptoms resistant to STN/GPi	Inconsistent results, technical targeting difficulties
**Cuneiform Nucleus**	Brainstem locomotor drive, glutamatergic	Freezing of gait, locomotion	More reliable than PPN; strong preclinical evidence	Still experimental; limited human data
**Centromedian–Parafascicular Complex**	Thalamostriatal pathway, attentional salience	Dyskinesias, refractory tremor, neuropsychiatric	Modulates motor + cognitive/limbic circuits	Pilot data only; not routine target
**Nucleus Basalis of Meynert**	Basal forebrain cholinergic projection	Cognitive impairment, attention, executive function	Potential benefit in PD dementia	Experimental, small studies, heterogeneous results
**Hypothalamus**	Autonomic regulation	Autonomic dysfunction (orthostatic hypotension, bladder)	Symptom-specific improvement	Very experimental; surgical/ethical risks
**Medial Forebrain Bundle**	Dopaminergic & limbic reward/motivation circuit	Apathy, depression, anhedonia	Promising for severe non-motor symptoms	Highly experimental; targeting precision issues

Legend: CM-Pf—Centromedian–Parafascicular Complex (thalamus), CnF—Cuneiform Nucleus, DRTt—Dentato-Rubro-Thalamic Tract, GPi—Globus Pallidus Internus, LEDD—Levodopa Equivalent Daily Dose, LHA—Lateral Hypothalamic Area, MFB—Medial Forebrain Bundle, NBM—Nucleus Basalis of Meynert, PH—Posterior Hypothalamus, PPN—Pedunculopontine Nucleus, PSA—Post-Subthalamic Area, SNr—Substantia Nigra pars Reticulata, STN—Subthalamic Nucleus, Vim—Ventral Intermediate Nucleus (of the thalamus), ZI/cZI—Zona Incertacaudal Zona Incerta.

## Data Availability

Not applicable.

## Abbreviations

CM-Pf	centromedian–parafascicular
CnF	cuneiform nucleus
cZI	caudal zona incerta
DBS	Deep brain stimulation
DRTt	dentato-rubro-thalamic tract
FOG	freezing of gait
GPe	external globus pallidus
GPi	internal globus pallidus
EFs	electric fields
MCI	mild cognitive impairment
MFB	medial forebrain bundle
MLR	mesencephalic locomotor region
NBM	nucleus basalis of Meynert
PD	Parkinson’s disease
PDD	Parkinson’s disease dementia
PPN	pedunculopontine nucleus
PSA	post-subthalamic area
RN	red nucleus
SNc	substantia nigra pars compacta
SNr	substantia nigra pars reticulata
STN	subthalamic nucleus
VA	ventral anterior nucleus
VIM	ventral intermediate nucleus
VL	ventral lateral nucleus
VM	ventral medial nucleus
VTA	volume of tissue activated
ZI	zona incerta
